# From click to green choice: the influence of social media content type on green consumption intention

**DOI:** 10.3389/fpsyg.2026.1652851

**Published:** 2026-05-08

**Authors:** Zhenzhen Peng, Ziduo Su, Mingfang Dong

**Affiliations:** School of Management, Xi’an University of Architecture and Technology, Xi’an, China

**Keywords:** behavioral agent type, greenconsumption intention, identity, information frame, interpretive hierarchy

## Abstract

**Purpose:**

This study focuses on the obstacles to the transformation of green consumption intention into behavior, explores the interaction between information framing (positive and negative) and behavioral agency types (individual and group), and reveals how two types of characteristics of social media video content influence consumers’ green consumption intention.

**Methods:**

Based on prospect theory, three scenario experiments were designed to simulate social media video stimuli. The study systematically manipulated two factors: information framing (emphasizing positive or negative outcomes) and behavioral agent types (presenting green behavior as either individual or group actions). Experiment 1 examined the interaction effect between these factors on green purchase intention; Experiment 2 explored the mediating role of self-identity and social identity; Experiment 3 investigated the moderating effect of explanatory level.

**Findings:**

This study investigates the interactive effects of information framing and behavioral agency types in social media video content on consumers’ green consumption intention. The results demonstrate that both positive information combined with individual behavior and negative information combined with group behavior significantly enhance green consumption intention. Self-identity and social identity mediate the impact of these two combinations, forming two distinct cognitive transmission pathways. Furthermore, the findings indicate that consumers’ explanatory level moderates the strength of this interactive effect, thereby altering how these two variables influence green consumption intention.

**Practical implications:**

This study provides targeted and actionable guidance for social media marketing managers, advertising professionals in the green product industry, and environmental governance departments: it proposes precise matching strategies of behavioral agent types and information frameworks, clarifies the identity activation mechanism to embed identity cues in green communication content, and formulates stratified communication strategies based on consumers’ interpretation levels and cognitive characteristics. The research conclusions can directly optimize the design of green social media content, improve the conversion efficiency of green consumption intentions to behavior, and provide a practical basis for the government to guide public green consumption behavior through digital media.

**Originality and value:**

The core contribution of this study lies in extending prospect theory to the context of green consumption communication in social media with empirical verification and mechanism exploration, and constructing a multi-variable synergistic influence model of “behavioral agent type-information framing-identity identification-level” on green consumption intention. It clarifies the differentiated cognitive transmission pathways of self-identity and social identity, and identifies the boundary condition of interpretation level for the interactive effect of behavioral agent type and information framing. This research not only expands the application boundaries of prospect theory, identity theory and interpretation level theory in the field of green consumption, but also provides actionable and stratified optimization strategies for green information dissemination, thereby promoting the widespread adoption of eco-friendly products and the realization of macro ecological governance goals.

## 1 Introduction

Under the dual impetus of achieving carbon neutrality and building an ecological civilization, consumers’ green consumption behaviors serve as the micro-foundation for promoting clean energy, reducing carbon emission intensity, and developing a circular economy, which holds critical significance for macro-level ecological governance and sustainable development (Testa et al., 2021). However, the “Citizen Ecological Behavior Survey Report (2020)” released by China’s Ministry of Ecology and Environment shows that while the public has a high level of recognition of the value of green consumption, only 50% of them actually practice it, indicating real obstacles in transforming green consumption intentions into actions (Sun and Miao, 2018). This intention-behavior gap has become a core bottleneck restricting the development of green consumption, and exploring its influencing factors and intervention paths has become a research hotspot in the field of sustainable marketing and environmental psychology.

The rapid proliferation of digital technology has transformed social media from a passive information tool into a powerful medium for shaping consumer cognition (Shen et al., 2020). Platforms like Bilibili and YouTube leverage massive user engagement to restructure environmental discourse through diverse content characteristics, such as information framing and behavioral agent types (Samarah et al., 2022). While existing research confirms the impact of social media on green consumption, most studies focus on isolated content features, leaving the synergistic mechanisms of multiple characteristics largely unexplored.

Central to this discourse is the heterogeneity of behavioral agent types—distinguishing between individual and group subjects—which significantly influences consumer identity and decision-making logic (Sheng et al., 2023). Previous studies have examined these agents through lenses such as self-transcendence (Graham and Abrahamse, 2017), perceived actionability (Olsson et al., 2020), and social class (Hwang and Moon, 2023). However, these studies have two obvious limitations: on the one hand, they overlook the critical variable of information framing, and fail to thoroughly analyze its interactive relationship with behavioral agency types or their synergistic effects on green consumption; on the other hand, they only focus on the direct effect of behavioral agency types, and lack an in-depth exploration of the psychological mediation mechanism linking behavioral agency types and green consumption intention.

According to Prospect Theory, individual decision-making relies on reference points such as current status or psychological expectations, leading to significant asymmetry in perceived gains and losses (Stewart et al., 2003), a principle that underpins the framing effect (Tversky and Kahneman, 1981). In green communication, the alignment between an agent (individual vs. group) and the message frame (positive vs. negative) may determine persuasive efficacy (Tversky and Kahneman, 1981; Aaker and Lee, 2001). This principle provides a crucial perspective for analyzing the effects of green communication in social media, revealing that the alignment between different types of actors (individuals/groups) and corresponding information framing may directly influence the effectiveness of stimulating green consumption intentions. Existing green consumption research on framing effects confirmed the differential effects of positive and negative information (Amatulli et al., 2019; Grappi et al., 2024), but the research gap lies in the lack of clarification on the matching context of framing effects, i.e., what kind of behavioral agent type can maximize the persuasive effect of positive/negative information, and what is the underlying psychological mechanism. For instance, environmental bloggers sharing personal practices like using bamboo fiber tableware instead of disposable plastic products can guide consumer imitation through positive messaging^1^. while documentaries exposing environmental issues like plastic pollution through negative framing have sparked social discussions, indirectly driving a surge in green consumption behacior^2^. These practical phenomena confirm the existence of a matching effect between behavioral agent types and information framing, but existing research has yet to systematically clarify the matching mechanism and its psychological transmission path.

Furthermore, the formation of consumers’ green consumption intention is not an isolated individual decision. It not only reflects personal values but is also deeply embedded in the process of social identity construction (Townsend and Sood, 2012). Individuals strengthen their self-identity and gain a sense of group belonging through green consumption choices, and this identity mechanism is further amplified in social media scenarios (Schulte et al., 2020; Becerra et al., 2023). Existing research has confirmed that self-identity and social identity are important predictors of green consumption behavioral (Whitmarsh and O’Neill, 2010; Zhang et al., 2023), but it remains unclear how different combinations of behavioral agent types and information framing activate self-identity and social identity in a differentiated manner, and whether identity plays a mediating role in the synergistic effect of the two variables. In addition, according to the level theory of interpretation, consumers exhibit heterogeneity affects the intensity and direction of their behavioral motivations, potentially leading users with different interpretation levels to react differently to the interaction between “behavioral agency types (individual/group) and information framing (positive/negative).”However, existing research has not explored the boundary condition of interaction level for the matching effect of behavioral agency types and information framing, which makes it impossible to formulate stratified green communication strategies for different cognitive characteristics of consumers.

Building on this foundation, this study focuses on behavioral agency types in social media, integrating prospect theory, identity theory, and explanatory level theory to systematically explore their influence mechanisms on green consumption intention. Specifically, the research aims to address three core questions: (1) clarifying the interactive effects between social media information frameworks and behavioral agency types on green consumption intention, and verifying the matching effect of “individual behavior-positive information” and “group behavior-negative information”; (2) revealing the differentiated mediating pathways of self-identity and social identity in these interactive effects; (3) testing the moderating role of user interpretation levels on the overall influence mechanism based on explanatory level theory. and identifying the cognitive boundary of the matching effect. The theoretical value of this study lies in elucidating the formation mechanisms of green consumption intention in social media contexts from the perspective of multi-variable synergy, expanding the application boundaries of prospect theory and identity theory in the field of green consumption. It provides scientific evidence for enterprises to optimize green marketing communication strategies, and for governments and non-profit organizations to guide public green consumption behaviors through social media, thereby supporting the realization of carbon neutrality and ecological civilization construction goals.

## 2 Theoretical basis and research hypotheses

### 2.1 Social media content types and green consumption

Social media is an online network encompassing digital platforms, applications, and websites, with its core function being to enable users to share and access content (Appel et al., 2020). It plays a significant role in reshaping consumer behavior by breaking the information asymmetry between enterprises and consumers, and constructing a social interaction context for consumption decision-making (Zhao et al., 2019; Alam et al., 2023) .In the field of green consumption, social media outperforms traditional media in delivering environmental content, with greater consumer appeal (Alsaad et al., 2023) on the one hand, it enhances the persuasiveness of environmental messaging through vivid video forms and interactive communication methods, further influencing consumers’ green product purchase decisions (Pittman et al., 2022; Shen et al., 2023); on the other hand, consumers who actively engage with social media green content are more likely to be motivated by green messaging, which in turn increases their attention to personal environmental practices and promotes the transformation of green cognition into behavior (Han et al., 2021). Confetto et al. (2023) further pointed out that sustainability content on social media can not only motivate consumers to adopt green consumption but also transform their lifestyles, forming a long-term green consumption habit.

However, existing literature has not yet answered a core question: as social media video content has diverse presentation forms, how do different combinations of content characteristics affect green consumption behavior? The core variable influencing this effect is the presentation form of the behavioral agent-whether the behavior is carried out by individuals or groups. Behavioral agent type is the basic premise for consumers to attribute environmental responsibility and perceive the consequences of consumption behavior (Sheng et al., 2019), and its matching with information framing directly affects the effectiveness of green information communication. Based on this, this study focuses on the types of behavioral agents in social media, and explores its interactive effect with information framing on consumers’ willingness to engage in green consumption, as well as the underlying psychological mechanism and boundary conditions.

#### 2.1.1 Types of behavioral agents and green consumption

Within the research framework of green consumption on social media, identifying the behavioral agents of information dissemination serves as the fundamental prerequisite for clarifying environmental responsibility attribution and defining the perceived consequences of consumption behaviors (Sheng et al., 2019). Existing studies have categorized social media actors based on different logical frameworks, such as stakeholders (businesses and consumers) (Kyu Kim et al., 2021) or organizational forms (individuals and groups) (Olsson et al., 2020). Given this study’s focus on the impact of green content on consumers, we adopt an individual-group subject classification framework to explore the role positioning and mechanisms of these two groups in disseminating green consumption concepts and guiding consumer behavior, because this classification is more in line with the cognitive habits of social media users in processing green information, and can directly reflect the difference in responsibility attribution and behavioral reference brought by different behavioral agents.

Extensive research has yielded substantial findings on green consumption behaviors at both individual and group levels, with particular attention paid to the influence of group dynamics. Studies confirm that group-level green action demonstrations and advocacy significantly enhance individuals’ willingness and likelihood to participate in green consumption (White et al., 2019), a phenomenon particularly pronounced in social responsibility-driven communication contexts (Fritsche et al., 2018), because group behavior can form social norms and trigger the psychological effect of “conformity” in consumers. However, the role of individual actors in green consumption communication remains equally vital: when communication focuses on the personal consequences of non-green consumption behaviors, consumers can more intuitively perceive the connection between their actions and ecological impacts, thereby strengthening their motivation to engage in green consumption (Aberman and Plaks, 2022). Practically speaking, individual green purchasing decisions serve as the “last mile” in translating ecological values into action (Steinhorst and Klöckner, 2018), acting as a unique intermediary that bridges macro-level ecological goals with micro-level consumption behaviors.

From a deeper logical perspective, the essence of green consumption lies in the balance and synergy between individual interests and collective ecological wellbeing (Fragnière, 2016; Hormio, 2023). The communication strategies of different behavioral agents in social media align with this logic: Green content dissemination centered on individuals often focuses on direct personal benefits like energy conservation and cost reduction, stimulating consumers’ self-interested participation motives (Luan et al., 2023). Conversely, group-oriented communication emphasizes social norm constraints and public environmental risks such as climate change, awakening consumers’ sense of collective responsibility (Luan et al., 2023). This fundamental difference in communication orientation means that consumers’ reception and feedback effects of environmental information will significantly diverge depending on the type of behavioral agent and the way information is presented. According to prospect theory, information is categorized as positive or negative. When using different behavioral agents in social media videos, selecting matching information frameworks is crucial to maximize effectiveness. Therefore, this study focuses on the interaction between “individual and group green behavior information” and “positive and negative information” in social media, and explores their synergistic effect on green consumption intention.

#### 2.1.2 Positive and negative information and green consumption

Prospect theory posits that decision-makers exhibit significantly greater sensitivity to perceived losses than equivalent gains, with this loss aversion tendency causing systematic differences in decision-making preferences between gain and loss framing (Stewart et al., 2003). The framing effect, a key derivative concept of this theory, specifically refers to how logically equivalent information triggers distinct psychological responses and behavioral choices when presented through different framing approaches (gain/loss orientation) (Tversky and Kahneman, 1981; Lee and Aaker, 2004). This phenomenon provides a crucial theoretical framework for understanding information dissemination mechanisms in the green consumption domain, because green consumption is a typical “cost-benefit trade-off” decision-making behavior, and consumers’ perception of the gains and losses of green consumption is directly affected by information framing.

In green consumption research, scholars typically categorize environmental information into positive and negative messages based on their environmental impact attributes. Positive messages highlight the benefits of green consumption, such as energy efficiency, cost reduction, and ecological improvement. These messages enhance consumers’ perceived behavioral efficacy, evoke positive emotions like pleasure and identification, and ultimately increase their willingness and inclination to participate in green consumption (Laskey et al., 1989; Stadlthanner et al., 2022; Grappi et al., 2024). The core mechanism of positive information is to reduce the perceived cost of green consumption and highlight its tangible benefits, thereby stimulating consumers’ active participation. Conversely, negative messages focus on environmental risks and behavioral losses, such as intensified climate disasters and ecosystem degradation. By triggering consumers’ loss aversion and moral negative emotions like guilt and shame, they create driving forces for environmental behavior under specific circumstances (Maheswaran and Meyers-Levy, 1990; Amatulli et al., 2019). The core mechanism of negative information is to enhance the perceived risk of non-green consumption and arouse consumers’ moral responsibility, thereby pushing them to engage in green consumption.

Notably, existing research on framing effects in green consumption remains inconclusive, with some studies failing to identify optimal scenarios for either positive or negative messaging (Ropret Homar and Knežević Cvelbar, 2021). Evidence suggests that combining both types of information effectively enhances communication persuasiveness by clearly demonstrating the benefits of green consumption while emphasizing the environmental costs of inaction. However, a critical research gap persists: the optimal contexts and target consumer groups for these framing approaches remain unclear. Particularly in social media environments where information is presented through different behavioral agents (individuals or groups), the mechanisms linking framing types to behavioral agents and their synergistic effects on green consumption intention require systematic exploration. This gap provides a key research focus for our study: we argue that the effect of information framing is not isolated, but is constrained by the type of behavioral agent, and only the matching combination of the two can maximize the persuasive effect of green information.

#### 2.1.3 Interaction between behavioral agent types and information framework

In the context of green consumption communication on social media, there are significant differences in the information focus and expression logic between individual and group subjects. Individual subjects primarily share personal experiences, specific details, and unique benefits derived from their green consumption choices, emphasizing the environmental attributes and personalized value of their eco-friendly decisions. The core message is that “individual actions can contribute to ecological protection,” highlighting the practicality and unique value of personal engagement. In contrast, group subjects focus on collaborative environmental initiatives such as community waste sorting, public welfare eco-projects, and industry-wide green campaigns. They highlight the widespread environmental improvements and social impacts generated by these collective efforts, conveying the ideas of “many hands make light work” and “united efforts to protect the ecosystem.” This approach underscores the advantages of group actions in achieving scale and strong influence.

According to prospect theory, in the context of individual green consumption behavior, positive framing typically emphasizes direct benefits of environmental protection, such as reducing carbon footprint, saving energy costs, and improving quality of life. These messages not only align with consumers’ self-interest but also enhance participation willingness through positive emotions like pride and satisfaction. For consumers with independent self-concept, green consumption serves as a means to demonstrate self-worth. Such consumers tend to focus on positive information related to themselves, especially when it highlights their personal interests. Therefore, when individual behavior types convey positive framing, they are more likely to perceive direct benefits of green consumption (e.g., reduced carbon emissions, cost savings), thereby strengthening their motivation to participate (Luan et al., 2023). In this scenario, positive framing reinforces self-efficacy, driving consumers to make sustainable development decisions. Thus, based on prospect theory, combining individual behavior types with positive framing can reduce consumers’ uncertainty and risks associated with green consumption by providing short-term, concrete benefits, thereby increasing willingness to engage. Consequently, when the video’s behavior type represents personal green actions, positive framing (compared to negative framing) proves more effective in boosting green consumption intention.

Compared to individual actions, group green behaviors emphasize collective social responsibility and normative expectations. The alignment between group behavior patterns and negative framing can significantly enhance consumers’ sense of social responsibility and group belonging. Negative framing typically highlights potential environmental degradation and social insecurity associated with non-green consumption, which effectively heightens consumers’ awareness of collective consequences. In group contexts, individuals are more susceptible to social norms, leading them to make decisions aligned with group expectations. Group behaviors prioritize collective interests over personal gains, prompting individuals to consider societal impacts rather than individual benefits when engaging in green consumption (Luan et al., 2023). When combined with negative framing, this approach emphasizes environmental risks, social accountability, and the necessity of collective action, effectively fostering social responsibility and encouraging participation in group efforts to mitigate adverse outcomes. This effect is particularly pronounced among group consumers, especially during environmental crises, where individuals tend to focus on leveraging collective power to prevent or mitigate ecological deterioration. Therefore, when the video’s behavioral type aligns with group green behavior, negative framing proves more effective than positive framing in boosting green consumption intention. Based on this analysis, the study proposes the following hypotheses:

*H1:* In social media videos, the interaction between behavioral agent types and information framing jointly influences consumers’ green consumption intention.

*H1a:* When the video’s behavioral agent type is individual green behavior, positive information (compared to negative information) is more effective in increasing green consumption intention.

*H1b:* When the video’s behavioral agent type is group green behavior, negative information (compared to positive information) proves more effective in boosting green consumption intention.

### 2.2 Mediating role of self-identity and social identity

Consumers’ green consumption decisions are not solely driven by information and subject types, but are deeply embedded in the process of identity construction and activation, where self-identity and social identity play key mediating roles in their interaction. Environmental psychology research has confirmed that self-identity, as a relatively stable psychological structure, can effectively predict individuals’ environment-related intentions and behaviors (Whitmarsh and O’Neill, 2010; Dean et al., 2012; Becerra et al., 2023). Essentially, it represents an individual’s self-awareness of their values, lifestyle habits, and core traits, which directly influences the value judgments in consumption decisions. Social identity reflects an individual’s self-definition as a member of a group sharing common values, goals, and norms, playing a pivotal role in guiding behavior within social contexts (Schulte et al., 2020). The formation and evolution of social identity increasingly drive individual behavior through group norms and societal expectations, particularly in areas involving collective goals such as environmental protection and green consumption (Steg, 2023). The core difference between the two identities lies in the focus of self-definition: self-identity focuses on individual characteristics and personal values, while social identity focuses on group membership and collective norms. This difference determines that the two identities will be activated in a differentiated manner under different combinations of behavioral agent types and information framing, and then mediate the influence on green consumption intention.

From the perspective of self-identity mediation, individuals tend to evaluate green information on social media by integrating personal goals, self-affirmation needs, and established behavioral patterns (Townsend and Sood, 2012). In videos highlighting personal actions, the positive impacts of individual behaviors are particularly emphasized, such as “adopting energy-saving measures can significantly reduce personal water and energy consumption while minimizing waste.” This type of information not only conveys the health benefits and cost-effectiveness of green consumption behaviors but also highlights associated values like energy conservation and sustainable development. Such messaging prompts consumers to reflect on the alignment between their actions and personal values, encouraging deeper contemplation of their environmental identity and responsibility for conservation, thereby activating environmental self-identity (Van Tonder et al., 2023). According to identity theory, individuals are more likely to accept information that aligns with their interests and achieves self-validation. Compared to negative messages emphasizing the adverse consequences of non-environmental behaviors and having weaker relevance to personal self-evaluation, positive green messages transmitted by individuals significantly strengthen self-identity. When environmental self-identity is activated and reinforced, consumers are more inclined to adopt behaviors consistent with this identity, thereby enhancing their willingness for green consumption.

From the perspective of social identity mediation, videos showcasing group behaviors, by conveying information aligned with social group interests, effectively reinforce individuals’ identification with group actions and values. When these videos emphasize the systemic nature of environmental issues and the importance of collective action, individuals recognize these challenges as not just personal but shared responsibilities. Such content typically highlights how group collaboration and collective efforts promote environmental sustainability, prompting viewers to reflect on the connection between their actions and group interests. When social media disseminates information consistent with group interests, individuals become aware of how their behaviors impact the collective, thereby fostering environmental responsibility. Notably, when negative consequences are associated with group behaviors, individuals more readily understand the adverse effects of non-green actions on society. This awareness deepens their sense of group identity and accountability. In evaluating and identifying negative information, understanding the detrimental impacts of non-green consumption behaviors further strengthens social group identification. Social identity theory posits that individuals who identify with a group are more likely to adopt its behavioral norms and adjust their own actions accordingly. In the context of green consumption, group members typically respond more actively to collective advocacy, comply with behavioral expectations, and contribute to sustainable practices through concrete actions (Zhang et al., 2023). This identification mechanism explains why individuals with stronger social identity tend to exhibit greater environmental awareness and adopt green consumption behaviors in daily life (Shen et al., 2010).

In summary, self-identity and social identity mediate through distinct subject-information alignment combinations. Based on this framework, the present study proposes the following hypotheses:

*H2:* For positive information, self-identity mediates the effect of individual behavior types on green purchase intention.

*H3:* Social identity mediates the effect of group behavior type video on green purchase intention for negative information.

### 2.3 Regulatory role of interpretation level

Differences in consumers’ interpretation levels of information lead to heterogeneous green consumption intentions and behavioral tendencies. While existing research acknowledges cognitive heterogeneity in processing complex information, there remains a lack of targeted exploration into how interpretation levels influence the promotion or inhibition of consumption intentions across different behavioral agents in social media contexts. The interpretation level theory posits that interpretation level refers to the degree of abstraction or concretization in an individual’s mental representation of information, and such cognitive differences directly impact cognitive judgment and decision-making processes (Trope and Liberman, 2010). Consumers with higher interpretation levels tend to think more abstractly and long-term, focusing on the long-term impacts and potential future benefits of issues. Conversely, those with lower interpretation levels prioritize immediate and tangible benefits, with their thinking patterns emphasizing short-term gains (Lee et al., 2010). This cognitive processing difference is the core basis for the moderating effect of interpretation level: different interpretation levels lead to different information processing preferences, and thus different responses to the combination of behavioral agent types and information framing.

This theory provides a robust framework for understanding how consumers process information and make decisions. Studies show that when information interpretation levels align with decision-making task requirements, it becomes more effective in activating and significantly influencing decisions, thereby enhancing their effectiveness and value. This phenomenon is known as the consistency effect of interpretation levels (Jin and He, 2013). In the context of green communication scenarios on social media examined in this study, green messages conveyed by different actors exhibit corresponding compatibility with two cognitive dimensions of explanatory levels. Specifically, green behaviors shared by individual actors are predominantly presented through perceptible daily life scenarios and practical operational details, accompanied by positive messaging emphasizing immediate benefits-aligning better with the cognitive preferences of low-explanatory-level individuals who focus on concrete and immediate information. Conversely, green initiatives advocated by group actors inherently possess stronger abstraction, emphasizing collective values and long-term ecological benefits, while negative messaging focusing on long-term risks tends to attract high-explanatory-level consumers.

This demonstrates that explanatory levels differently modulate the relationship between “behavioral agent type-information framing” interactions and green consumption intention: low-explanatory-level consumers respond more positively to individual actors and positive messaging, whereas high-explanatory-level consumers are more susceptible to negative messaging from group actors. For combinations where actors and messages are mismatched, no significant differences in consumer responses are observed across explanatory levels. In summary, explanatory levels primarily influence the overall impact mechanism by affecting consumers’ cognitive compatibility with different actor-information combinations. Based on these findings, this study proposes the following moderating effect hypotheses:

H4. Explanatory level moderates the impact of the interaction effect between behavioral agent types and information framing in social media on green consumption intention.

*H4a:* For consumers with low explanatory levels, positive information significantly enhances green consumption intention in individual-level green behavior scenarios compared to negative information, whereas no significant difference is observed between positive and negative information in group-level green behavior scenarios.

*H4b:* For consumers with high explanatory levels, negative information significantly enhances green consumption intention in group-level green behavior scenarios, whereas positive information shows no significant difference in individual-level scenarios.

**FIGURE 1:**
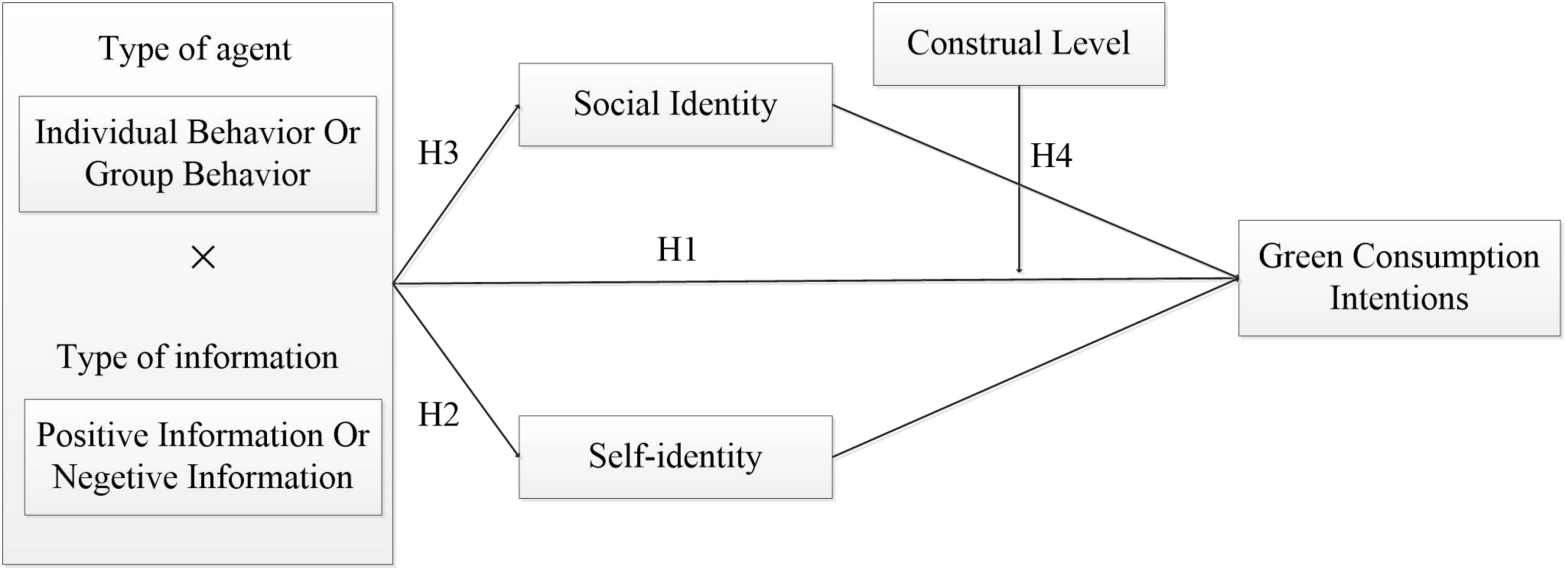
Theoretical framework.

In summary, this paper proposes the following theoretical framework, as shown in Figure 1.

## 3 Methodology

### 3.1 Preliminary experiment

To investigate how the interaction between video information framing and behavioral agent types influences green consumption intention, this study employed a questionnaire survey for data collection. The experimental design utilized a two-way between-subjects design (2: Information Framing: Positive vs. Negative × 2: Behavioral Agent Type: Individual vs. Group). To examine the interaction mechanism between information framing and behavioral agent types, a pilot experiment was conducted to screen suitable experimental video materials for the formal questionnaire survey, ensuring the validity and operability of the experimental stimuli.

#### 3.1.1 Sampling method for preliminary experiment

The participants of the preliminary experiment were recruited through the Credamo online research platform, which is a professional academic research sampling platform with a sample pool covering different age, gender, education and regional groups, and the sample quality is guaranteed by strict screening mechanisms (e.g., attention check questions, response time control). A total of 35 participants were recruited in this preliminary experiment, including 16 males (45.71%) and 19 females (54.29%), aged 18–55 years, with a college degree or above accounting for 88.57%. The sample size is determined based on the principle of exploratory pre-experiment sampling (generally 30–50 samples), which is sufficient to complete the screening of experimental video materials and the preliminary test of variable manipulation.

#### 3.1.2 Scale development for preliminary experiment

The scale for evaluating the relevance of video interactive content was developed based on the research objectives and existing literature, with reference to the scale development method of Sun and Miao (2018). The scale includes 4 items to measure the types of behavioral agents and information framing, using a 7-point Likert scale (1 = completely inconsistent, 7 = completely consistent). The specific items are: (1) Do you think the video content contains positive information rather than negative information? (2) Do you think the video content contains negative information rather than positive information? (3) Do you think the subject of the video is an individual rather than a group? (4) Do you think the subject of the video is a group rather than an individual? Before the formal investigation, the scale was pretested with 10 participants, and the items were revised for expression clarity, ensuring the face validity of the scale.

#### 3.1.3 Experimental stimulus material selection

When selecting experimental stimulus materials, Bilibili was chosen as the primary video source platform. First, Bilibili is one of China’s leading online video platforms, boasting a broad and diverse user base, including rich group types, which can ensure the representativeness of the experimental stimuli. Second, the content library of Bilibili is extensive, covering a large number of high-quality videos related to environmental protection with clear themes and diverse forms, which provides ample material sources for this study.

We carefully selected 12 environmental-themed videos from online video platforms (as shown in Table 1), with the selection criteria including: (1) the theme is closely related to green consumption and environmental protection, with a clear core message; (2) the content is clear and understandable, with no ambiguous expression of behavioral agent type and information framing; (3) the video duration is moderate (1–5 min), avoiding the influence of excessive duration on participants’ attention and judgment. To assess the relevance of interactive content in the videos, this study chose to conduct analysis through questionnaire surveys rather than collecting real data from the Bilibili platform, avoiding interference from the randomness of user behavior or uncontrollable data on experimental results.

**TABLE 1:** Overview of videos.

Video frequency	Information frame	Agent type	Video length
Video 1	Positive	Group	7 min and 27 S
Video 2	Positive	Group	6 min and 33 s
Video 3	Positive	Group	3 min and 2 s
Video 4	Positive	Individual	4 min and 15 s
Video 5	Positive	Individual	9 min and 2 s
Video 6	Positive	Individual	6 min and 43 s
Video 7	Negative	Individual	1 min and 32 s
Video 8	Negative	Individual	2 min and 44 s
Video 9	Negative	Group	5 min and 26 s
Video 10	Negative	Group	1 min and 52 s
Video 11	Negative	Group	2 min and 42 s
Video 12	Positive	Group	14 min and 12 s

#### 3.1.4 Preliminary experiment results and manipulation check

The 35 participants were asked to rate the relevance of interactive information for each video using the 7-point Likert scale. The manipulation check results showed that the average scores of the 4 items for each video were all above 4 (the midpoint of the scale), indicating that the participants could clearly perceive the behavioral agent type and information framing of the video content, and the manipulation of the experimental variables was effective. The specific relevance evaluation results were: 71.4, 71.4, 94.3, 91.4, 100, 94.3, 71.4, 60, 97.1, 82.9, 71.4, and 91.4% of participants believed these videos contained relevant interactive information. Based on these survey results, videos 3, 4, 7, and 10 were selected for the formal experiment due to their high relevance of interactive information, moderate duration, and clear variable expression.

### 3.2 Experiment 1

#### 3.2.1 Experimental materials

Experiment 1 aimed to verify how behavioral agent types and information framing jointly influence consumers’ green purchase intention, specifically testing Hypothesis 1 (H1). The study employed a two-way between-subjects factorial design with 2 factors (behavioral agent type: individual vs. group) × 2 factors (information framing: positive vs. negative).

##### 3.2.1.1 Sampling method

Participants were recruited through the Credamo professional academic research platform, with the sampling criteria including: (1) having the experience of browsing environmental protection or green consumption related content on social media; (2) being 18 years old or above, with independent consumption decision-making ability; (3) being able to complete the questionnaire and video viewing independently. A total of 200 participants were initially recruited, and the sample size is determined based on the sample size calculation formula for between-subjects experimental design (each group requires at least 30 samples, 4 groups require at least 120 samples), with an appropriate expansion to avoid invalid samples. After excluding invalid samples (3 cases with identical scores, 4 cases with abnormally short response times, and 12 cases with contradictory answers), 181 valid samples were retained. Following the attention check item (“Please select ‘strongly agree’ for this item”), 5 participants failed, resulting in a final valid sample size of 176 (validity rate 88%). The sample composition included 75 males (42.61%) and 101 females (57.39%), with age ranging from 18 to over 60 years, and education level. The sample structure is consistent with the user characteristics of social media video platforms, ensuring the external validity of the experiment.

#### 3.2.2 Experimental materials and scale development

##### 3.2.2.1 Experimental stimulus materials

The stimuli for Experiment 1 were social media videos sourced from the Bilibili platform, which provided environmental protection information. Based on the preliminary experiment results, videos 3, 4, 7, and 10 were used to create stimulus materials for four experimental scenarios. All four experimental scenarios presented the stimulus materials in a consistent layout (video viewing area+questionnaire answering area) while varying the information framing and behavioral agent types, to avoid the influence of layout differences on the experimental results. The content of the stimulus materials is detailed in Table 2.

**TABLE 2:** Introduction to experimental materials.

Group	Material content
Individual behaviora× ehaviora information	“For the sake of environmental sustainability, I choose more products, which not only saves resources and reduces pollution, but is also very convenient and beneficial to individuals.”
Individual behaviora× ehaviora information	“My environmentally irresponsible actions have exacerbated the environmental problems, the small fish they raise cannot survive, and their own lives have been disrupted.”
Group behavioro× ehavioro message	“Even though we face the challenge of environmental and climate issues, if we start to act and choose to move forward, if we stick together, we will be able to tackle this problem in the future.”
Group behavioru× ehaviorugh we face t	“Facing environmental problems, we always think we have time, but in reality, we are the first generation to know that we are destroying the world, and the last generation that can make a difference. Let’s fight together to save the world.”

##### 3.2.2.2 Scale development and reliability/validity test

All scales adopted in Experiment 1 are mature scales in existing literature with good reliability and validity, and appropriate revisions were made according to the research context of social media green consumption to ensure the content validity of the scales. All scales use a 7-point Likert scale (1 = strongly disagree, 7 = strongly agree).

(1) Green Purchase Intention Scale: Adopted from Srivastava and Gupta (2023), the scale includes 4 items, such as “After watching this video, I plan to spend more money on eco-friendly products rather than traditional products; I will consider products that cause less environmental pollution in the future” et al. The reliability of the green purchase intention scale was acceptable (Cronbach’s α = 0.759), and all constructs demonstrated composite reliability (CR) values above 0.7 and average variance extracted (AVE) values above 0.5, indicating good reliability and convergent validity.
(2) Information Framing Manipulation Scale: Referenced from Millstein et al. (2019), the scale includes 8 items, 4 for positive information framing (e.g., “These messages make me optimistic about environmental challenges,” “These messages make me optimistic about the future of the environment”) and 4 for negative information framing (e.g., “These messages make me worried about environmental problems,” “These messages make me feel the urgency of environmental protection”). The pretest results showed that the Cronbach’s α coefficients of the positive and negative dimensions were 0.815 and 0.832, respectively, indicating good internal consistency reliability, and all constructs demonstrated composite reliability (CR) values above 0.7 and average variance extracted (AVE) values above 0.5, indicating good reliability and convergent validity.
(3) Behavioral Agent Type Manipulation Scale: Drew from Wang and Howell (2010), the scale includes 8 items, 4 for individual behavior (e.g., “These contents motivate me to set environmental goals for myself,” “These contents enabled me to examine environmental issues from the perspective of ‘self-behavior”’) and 4 for group behavior (e.g., “These sections discuss the tasks our ‘community’ must accomplish for environmental protection purposes,” “These contents encourage our ‘community’ to work together toward an environmental protection goal”). The pretest results showed that the Cronbach’s α coefficients of the individual and group dimensions were 0.802 and 0.818, respectively, indicating good internal consistency reliability, and all constructs demonstrated composite reliability (CR) values above 0.7 and average variance extracted (AVE) values above 0.5, indicating good reliability and convergent validity.

In addition, the questionnaire also included demographic information such as age, gender, and education level, to control the influence of demographic variables on the experimental results.

#### 3.2.3 Experimental procedures

The experiment was conducted online through the Credamo platform, and the experimental procedures were strictly controlled to ensure the internal validity of the experiment. The specific steps are as follows: ① Informed Consent: Participants were presented with the experimental informed consent, including the research purpose, experimental process, time required, and confidentiality commitment, and only participants who agreed to the informed consent could enter the next step. ② Video Viewing: Participants were randomly assigned to one of the four experimental groups, and were required to watch the corresponding environmental protection video stimulus material. The platform set the video viewing time to ensure that participants watched the entire video. ③ Scale Measurement: After viewing the video, participants completed the Green Purchase Intention Scale, Information Framing Manipulation Scale, and Behavioral Agent Type Manipulation Scale in sequence. ④ Manipulation Check and Attention Test: The manipulation check items were embedded in the scale measurement, and an attention check item was set at the end of the questionnaire to screen out invalid samples. ⑤ Demographic Information Collection: Participants completed the demographic information questionnaire, including age, gender, education level, etc. ⑥ Reward and Debriefing: Participants who completed the questionnaire received corresponding cash rewards, and the research purpose and experimental design were briefly explained to the participants.

#### 3.2.4 Experimental results

##### 3.2.4.1 Manipulation check

Manipulation testing was conducted to verify whether the participants could correctly perceive the information framing and behavioral agent type of the experimental stimuli, and the results are presented as text analysis as follows:

First, an independent samples *t*-test was conducted to evaluate the success of the framing manipulation. The first four items of the framing manipulation check were averaged to form a positive index, while the last four items were averaged to form a negative index. The results of the ANOVA showed that participants in the individual behavior × positive information group perceived a significantly higher positive index than negative index for the video information [M_*pos*_ = 6.119, SD = 0.567; M_*neg*_ = 2.890, SD = 1.509; *t*(90) = 19.076, *p* < 0.01]. Similarly, participants in the group behavior × negative information group perceived a significantly higher negative index than positive index for the video information [M_*pos*_ = 2.078, SD = 0.698; M_*neg*_ = 5.366, SD = 1.478; *t*(96) = −19.193, *p* < 0.01]. The results indicate that the participants could clearly distinguish between positive and negative information framing, and the manipulation of information framing was effective.

Second, a manipulation check was performed on the agent type. The results showed that participants in the individual behavior group perceived higher individual behavior in videos describing individual behavior compared to group behavior [M_*indiv*_ = 6.125, SD = 0.585; M_*group*_ = 5.013, SD = 1.579; *t*(92) = 6.338, *p* < 0.01]. Participants in the group behavior group also perceived a higher group behavior index in videos depicting group behavior compared to those depicting individual behavior [M_*indiv*_ = 4.519, SD = 1.881; M_*group*_ = 6.159, SD = 0.446; *t*(94) = −8.223, *p* < 0.01]. The results indicate that the participants could clearly distinguish between individual and group behavioral agent types, and the manipulation of behavioral agent type was effective and consistent with expectations.

##### 3.2.4.2 The interaction effect between information framing and behavioral type

**FIGURE 2:**
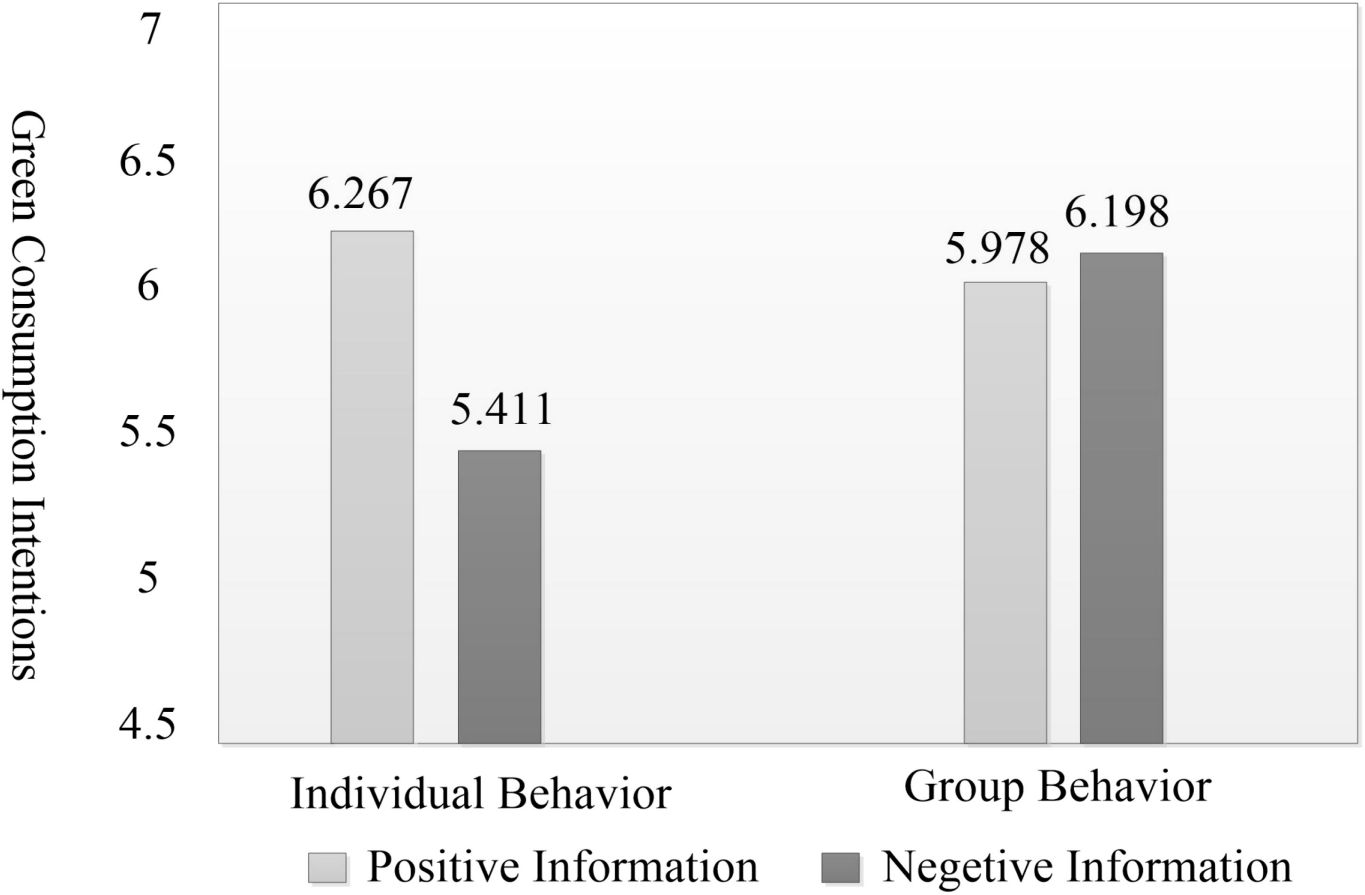
Interactive effect of behavioral agent type and information framework on green purchase intention.

Results from the two-way ANOVA showed that videos describing group behavior were more effective than those describing individual behavior in stimulating participants’ green purchase intention (M_*group*_ = 6.088, SD = 0.065; M_*indiv*_ = 5.839, SD = 0.064; *F*(1, 181) = 7.450, *p* < 0.01). Compared to negative information videos, positive information more effectively triggered participants’ proactive green purchase intention (M_*pos*_ = 6.122, SD = 0.066; M_*neg*_ = 5.805, SD = 0.063; *F*(1, 181) = 12.170, *p* < 0.01). As shown in Figure 2, there was a significant interaction effect between information framing and agent type [*F*(1, 181) = 34.877, *p* < 0.01].

To further elucidate the nature of this interaction effect, a simple effect analysis was conducted. The results demonstrated that when videos depicted individual behaviors, positive environmental information significantly outperformed negative environmental information in stimulating participants’ green purchase intention [M_*pos*_ = 6.267, SD = 0.131; M_*neg*_ = 5.411, SD = 0.091; *F*(1, 181) = 16.310, *p* < 0.05], which supports H1a. Conversely, when videos portrayed group behaviors, negative environmental information elicited more pronounced green purchase intention than positive information [M_*pos*_ = 5.978, SD = 0.131; M_*neg*_ = 6.198, SD = 0.091; *F*(1, 181) = 16.310, *p* < 0.05], which supports H1b. In summary, Hypotheses H1, H1a, and H1b are all validated.

#### 3.2.5 Discussion

Experiment 1 demonstrated the interaction effect between behavioral agent type and information framing on green purchase intention through rigorous experimental design and data analysis, with the manipulation of all experimental variables being effective and the measurement scales having good reliability and validity. The results confirm that when conveying individual behavioral information, positive environmental cues significantly enhanced consumers’ purchase intention; conversely, negative environmental cues promoted higher green purchase intention when communicating group behavioral information. This finding verifies the matching effect of behavioral agent type and information framing proposed in the research hypothesis, and lays a foundation for the subsequent exploration of the mediating mechanism and boundary conditions. Experiment 2 will further investigate the interaction between behavioral agent type and information framing, while exploring their potential mediating mechanisms of self-identity and social identity, and conduct more in-depth reliability and validity tests on the mediating variable scales.

### 3.3 Experiment 2

#### 3.3.1 Experimental design, scale development and experimental procedures

##### 3.3.1.1 Experimental design

Experiment 2 aimed to examine how the interaction between information framing and behavioral agent types influences consumers’ green purchase intention through the mediating effects of social identity and self-identity. Building upon the design framework of Experiment 1, this study introduced new measurement dimensions for social identity and self-identity, and adopted the same two-way between-subjects factorial design (2 behavioral agent type × 2 information framing).

##### 3.3.1.2 Scale development and reliability/validity test

On the basis of Experiment 1, Experiment 2 added the measurement of self-identity and social identity, and all new scales are also mature scales in existing literature with appropriate revisions according to the research context. All scales use a 7-point Likert scale, and the pretest and reliability/validity test results are as follows:

(1) Self-identity Scale: Adapted from Harrigan et al. (2018) and revised to fit the green consumption context, this scale consists of 4 items, including “I identify with the environmental behavior of the individual in the video,” “The environmental behavior in the video is consistent with my personal values,” “I am willing to imitate the environmental behavior of the individual in the video,” and “My environmental awareness is consistent with that of the individual in the video.” A pretest was conducted on 60 participants, yielding a Cronbach’s α coefficient of 0.909, which indicates excellent internal consistency reliability. Additionally, the average variance extracted (AVE) was 0.76 and the composite reliability (CR) was 0.91, both demonstrating good convergent validity.

(2) Social Identity Scale: Adapted from Cameron (2004) and adjusted to align with the green group behavior context, this scale consists of 4 items, including “I identify with the environmental behavior atmosphere of the group in the video,” “I am willing to be a member of the environmental protection group in the video,” “The environmental values of the group in the video are consistent with mine,” and “I support the collective environmental behavior in the video.” A pretest was administered to participants, with the results showing a Cronbach’s α coefficient of 0.962, indicating an excellent level of internal consistency reliability. The average variance extracted (AVE) was 0.85 and the composite reliability (CR) was 0.96, which demonstrated good discriminant validity.

##### 3.3.1.3 Experimental procedures

Participants were recruited from the Credamo platform and randomly assigned to four experimental groups. After excluding ineligible samples, 180 valid samples were retained, comprising 75 males (41.67%) and 105 females (58.33%). Experiment 2 adopted the same online experimental procedure as Experiment 1, with minor adjustments to the scale measurement link to add the self-identity and social identity scales.

#### 3.3.2 Experimental results

##### 3.3.2.1 Manipulation check

Manipulation checks for information framing and behavioral agent type were conducted first, with all tests using independent samples *t*-test and ANOVA, and the results are integrated into text analysis as follows:

##### 3.3.2.2 Information framing manipulation

For the individual behavior group, participants perceived significantly higher positive information framing scores in the positive information subgroup than in the negative information subgroup [M_*pos*_ = 5.925, SD = 0.903; M_*neg*_ = 2.925, SD = 1.443; *t*(93) = −20.666, *p* < 0.01]. For the group behavior group, the negative information subgroup had significantly higher negative framing scores than the positive information subgroup [M_*pos*_ = 2.610, SD = 1.084; M_*neg*_ = 5.871, SD = 1.029; *t*(87) = 16.582, *p* < 0.05]. The above results show that the manipulation of the information framing variable is consistent with the research design expectations, and the manipulation is effective.

##### 3.3.2.3 Behavioral agent type manipulation

Participants in the individual behavior group perceived a significantly higher individual behavior index in the video than the group behavior index [M_*indiv*_ = 6.163, SD = 0.634; M_*group*_ = 4.518, SD = 1.785; *t*(83) = 8.983, *p* < 0.01]. Participants in the group behavior group perceived a significantly higher group behavior index than the individual behavior index [M_*indiv*_ = 3.889, SD = 1.857; M_*group*_ = 6.201, SD = 0.470; *t*(97) = −10.664, *p* < 0.01], This indicates that the behavioral agent type in the experimental stimuli was clearly perceived by participants, and the manipulation was consistent with the research design expectations.

##### 3.3.2.4 Interaction effect test of information framing and behavioral agent type

General linear model analysis was used to test the interaction effect, and the results showed a significant interaction effect between behavioral agent type and information framing on green purchase intention [*F*(1, 179) = 60.907, *p* < 0.01]. Further simple effect analysis showed that when the video described individual behavior, positive information framing significantly improved participants’ green purchase intention compared with negative information framing [M_*pos*_ = 6.168, SD = 0.630; M_*neg*_ = 5.493, SD = 0.919; *F*(1, 82) = 15.674, *p* < 0.01], which further verified H1a. When the video depicted group green behavior, negative information framing triggered a significantly higher green purchase intention than positive information framing [M_*pos*_ = 5.207, SD = 1.010; M_*neg*_ = 6.450, SD = 0.331; *F*(1, 96) = 67.957, *p* < 0.01]. which further verified H1b. The above results reconfirmed the Hypothesis H1, and the interaction effect was more significant than that in Experiment 1, indicating the stability and robustness of the matching effect between behavioral agent type and information framing.

##### 3.3.2.5 Mediating effect test of self-identity and social identity

Using Model 8 in the PROCESS macro program, with 5000 bootstrap samples and a 95% confidence interval, the mediating effects of self-identity and social identity were analyzed, with green purchase intention as the dependent variable, behavioral agent type and information framing as independent variables, and gender and age as control variables. The test results are as follows:

*Mediating effect of self-identity*: The bootstrap 95% confidence interval of the mediating effect was (0.140–0.298), which did not include 0, indicating that self-identity had a significant mediating effect on the interaction between behavioral agent type and information framing on green purchase intention. Further grouping analysis found that in the individual behavior group, the mediating effect of self-identity was significant [95% CI (−0.218 to −0.053), excluding 0]; in the group behavior group, the mediating effect was not significant [95% CI (−0.076 to 0.234), including 0]. This confirmed that self-identity only mediates the influence of individual behavior+positive information on green purchase intention, and Hypothesis H2 was supported.

*Mediating effect of social identity*: The bootstrap 95% confidence interval of the mediating effect was (0.023–0.171), which did not include 0, indicating that social identity had a significant mediating effect. Grouping analysis showed that in the group behavior group, the mediating effect of social identity on group behavior+negative information was significant [95% CI (0.012–0.189), excluding 0]; in the individual behavior group, the mediating effect was not significant [95% CI (−0.093 to 0.092), including 0]. This confirmed Hypothesis H3.

**FIGURE 3:**
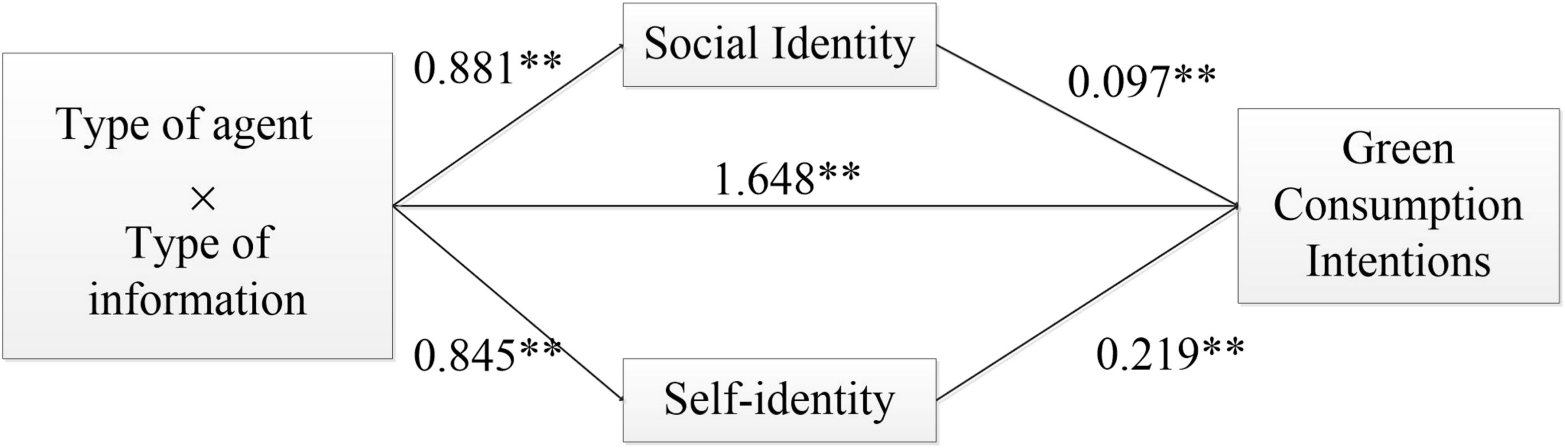
Mediating path of interaction effect between behavioral agent type and information framework. *p < 0.05, **p < 0.01, and ***p < 0.001.

The specific mediating path coefficients are shown in Figure 3, and all direct and indirect effects were significant at the *p* < 0.05 level, which clarified the differentiated mediation mechanism of the two identity variables.

#### 3.3.3 Discussion

Experiment 2 reaffirmed the interaction effect between behavioral agent types and information framing on green consumption intention through a larger sample size and more rigorous scale measurement. More importantly, this experiment verified the differentiated mediating role of self-identity and social identity in the above interaction effect: self-identity is the cognitive transmission path of “individual behavior+positive information,” while social identity is the cognitive transmission path of “group behavior+negative information.”

This finding reveals the intrinsic psychological mechanism of the matching effect between behavioral agent type and information framing: positive information of individual behavior activates consumers’ environmental self-identity by highlighting the consistency between personal behavior and self-values, and then promotes green purchase intention; negative information of group behavior enhances consumers’ sense of group belonging and social responsibility by emphasizing the collective consequences of non-green consumption, and social identity further translates this psychological perception into consumption intention. At the same time, the good reliability and validity of all scales ensure the scientificity of the research results, laying a foundation for the subsequent exploration of the moderating effect of interpretation level.

### 3.4 Experiment 3

#### 3.4.1 Experimental design and sampling method

##### 3.4.1.1 Experimental design

Experiment 3 primarily aimed to achieve two research objectives: first, to retest hypotheses H1a, H1b, H2, and H3 with a larger sample to verify the robustness of the research conclusions; second, to examine the moderating effect of interpretation level on the interaction between behavioral agent type and information framing (Hypotheses H4, H4a, and H4b). The experiment adopted a three-way between-subjects factorial design: 2 (behavior type: individual vs. group) × 2 (information framing: positive vs. negative) × 2 (interpretation level: high vs. low).

##### 3.4.1.2 Sampling method

Participants were recruited from the Credamo professional academic research platform, with the same basic sampling criteria as Experiments 1 and 2 (having social media green content browsing experience, 18 years old or above, with independent consumption decision-making ability). In addition, considering the three-way factorial design, the sample size was expanded according to the sample size standard of multi-factor experimental design (at least 30 samples per group, 8 groups in total require at least 240 samples). A total of 320 participants were initially recruited, and after excluding invalid samples (12 cases with abnormally short response times, 9 cases with contradictory answers, 10 cases failing the attention check and interpretation level manipulation check), 289 valid samples were finally retained (validity rate 90.31%).

#### 3.4.2 Experimental materials and scale development

##### 3.4.2.1 Experimental stimulus materials

The environmental protection video materials of behavioral agent type and information framing were consistent with Experiments 1 and 2 (Videos 3, 4, 7, 10 from Bilibili). The manipulation of interpretation level was based on the classic experimental method of Sheng et al. (2019), with “improving and maintaining physical health” as the priming theme, and two sets of thinking exercises were designed to manipulate the high and low interpretation levels of participants.

##### 3.4.2.2 Scale development and reliability/validity test

On the basis of Experiments 1 and 2, Experiment 3 added the Interpretation Level Scale (adopted from Trope and Liberman, 2010, revised for the Chinese context), which included 10 items (e.g., “I tend to think about the long-term meaning of things,” “I pay more attention to the specific implementation steps of things”), using a 7-point Likert scale (1 = strongly disagree, 7 = strongly agree). The scale was divided into two dimensions: high interpretation level (5 items) and low interpretation level (5 items).

Pretest was conducted with 70 participants, and the results showed that the Cronbach’s α coefficient of the whole scale was 0.784, the high interpretation level dimension was 0.769, and the low interpretation level dimension was 0.772, all meeting the reliability requirements. AVE = 0.52, CR = 0.78, indicating good construct validity.

#### 3.4.3 Experimental procedures

Experiment 3 adopted an online experimental procedure, and the specific steps were optimized on the basis of the previous two experiments to add the interpretation level manipulation link, with strict control of the experimental process to avoid confounding variables: Participants were randomly assigned to the high or low interpretation level group, and completed the corresponding thinking exercises. High interpretation level group: Answer the question “Why improve and maintain physical health?” and three consecutive “why” follow-up questions to guide abstract and long-term thinking. Low interpretation level group: Answer the question “How to improve and maintain physical health?” and three consecutive “how” follow-up questions to guide concrete and immediate thinking. Participants completed the Interpretation Level Scale to test the effectiveness of the manipulation. Participants were randomly assigned to one of the four behavioral agent type × information framing groups, and watched the corresponding green video materials. Completed the Green Purchase Intention Scale, Self-identity Scale, Social Identity Scale, Information Framing Manipulation Scale and Behavioral Agent Type Manipulation Scale in sequence.

#### 3.4.4 Experimental results

##### 3.4.4.1 Manipulation check

Independent samples *t*-test showed that the interpretation level score of the high interpretation level group was significantly higher than that of the low interpretation level group [M_low_ = 4.83, M_high_ = 7.04, *F*(1, 287) = 4.058, *p* < 0.05]), indicating that the manipulation of interpretation level was effective, and participants formed corresponding cognitive thinking modes as expected.

##### 3.4.4.2 Retest of interaction effect and mediating effect

Interaction effect retest: Two-way ANOVA showed that the interaction effect between behavioral agent type and information framing on green purchase intention was still significant [*F*(1, 288) = 45.767, *p* < 0.05]. Simple effect analysis reconfirmed H1a and H1b: individual behavior with positive information significantly improved green purchase intention (*p* < 0.05), and group behavior with negative information had a more significant promoting effect (*p* < 0.05).

##### 3.4.4.3 Mediating effect retest

Bootstrap test with 5,000 samples showed that the mediating effect of self-identity was significant [95% CI (0.236–0.360), excluding 0], and it was only significant in the individual behavior group (*p* < 0.01); the mediating effect of social identity was significant [95% CI (0.088–0.179), excluding 0], and it was only significant in the group behavior group (*p* < 0.05). Hypotheses H2 and H3 were reaffirmed, indicating that the research conclusions have good robustness.

##### 3.4.4.4 Moderating effect test of interpretation level

Three-way ANOVA was used to test the moderating effect of interpretation level, with green purchase intention as the dependent variable, behavioral agent type (A), information framing (B) and interpretation level (C) as independent variables. The results showed that the three-way interaction effect of A × B × C was significant [*F*(1, 288) = 4.744, *p* < 0.05], which verified Hypothesis H4 that interpretation level moderates the interaction between behavioral agent type and information framing.

Further simple effect analysis was conducted to explore the specific moderating effect, and the results supported H4a and H4b: For low interpretation level consumers (focus on concrete and immediate benefits): In the individual behavior scenario, positive information significantly enhanced green purchase intention compared with negative information [M_pos_ = 6.462, SD = 0.519; M_neg_ = 5.141, SD = 0.729; *F*(1, 64) = 71.120, *p* < 0.05]; in the group behavior scenario, there was no significant difference between positive and negative information [*F*(1, 64) = 0.637, *p* > 0.05] (Hypothesis H4a supported). For high interpretation level consumers (focus on abstract and long-term impacts): In the group behavior scenario, negative information significantly improved green purchase intention compared with positive information [M_neg_ = 6.378, SD = 0.347; M_pos_ = 5.858, SD = 0.694; *F*(1, 73) = 16.657, *p* < 0.05]; in the individual behavior scenario, there was no significant difference between positive and negative information [*F*(1, 64) = 3.509, *p* > 0.05] (Hypothesis H4b supported).

**FIGURE 4:**
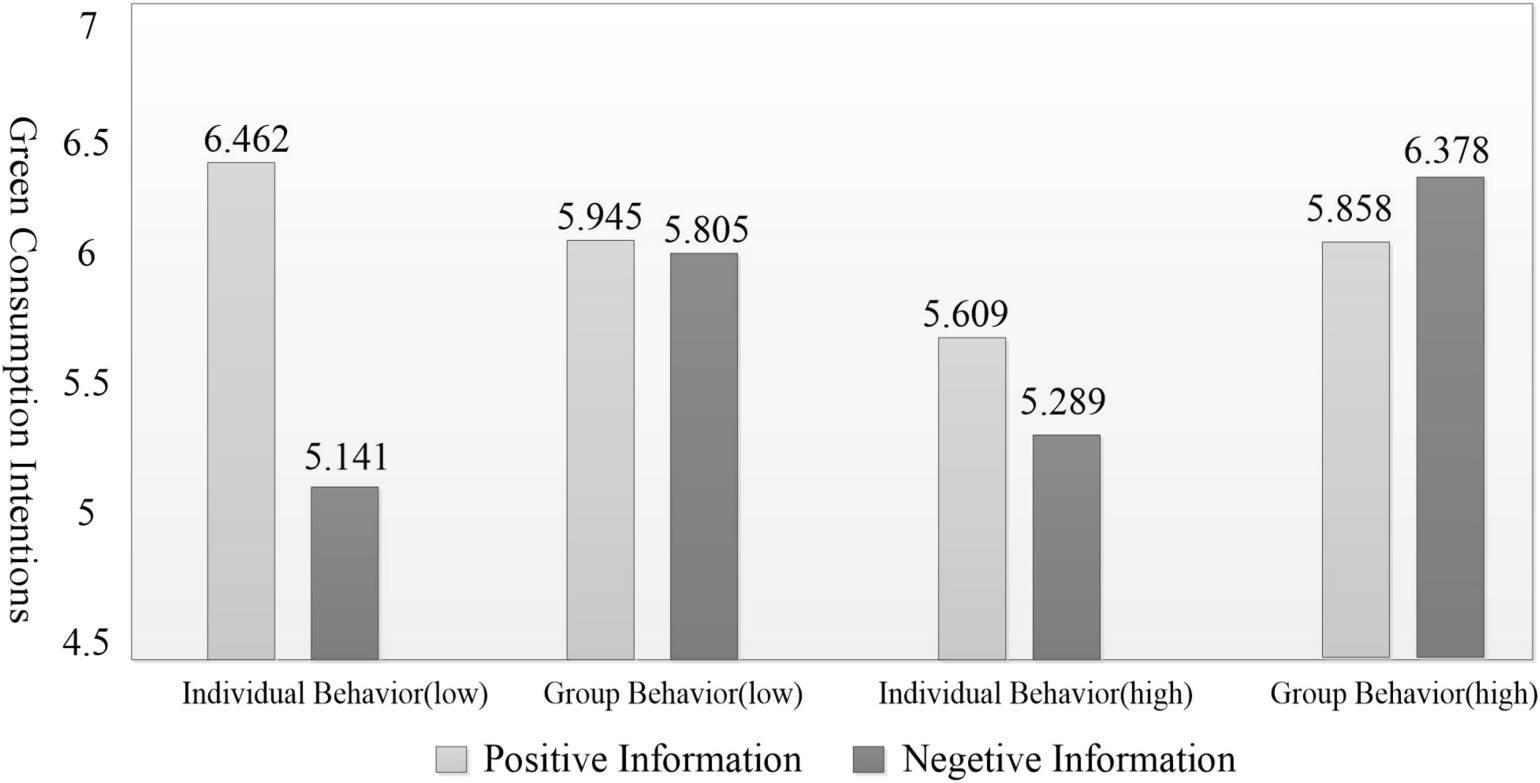
Illustrates the moderating effect of explanatory level on the interaction between behavioral agent type and information frame.

The specific moderating effect trend is shown in Figure 4, which clearly reflects the cognitive matching effect between interpretation level and the combination of behavioral agent.

#### 3.4.5 Discussion

Experiment 3 completed three core research tasks through a rigorous three-way between-subjects experimental design: first, retested the interaction effect between behavioral agent type and information framing with a larger sample, and verified the robustness of the matching effect of “individual behavior + positive information” and “group behavior + negative information”; second, reconfirmed the differentiated mediating role of self-identity and social identity, and further verified the stability of the cognitive transmission mechanism; third, for the first time, revealed the moderating effect of interpretation level on the above interaction effect, and clarified the cognitive boundary condition of the matching effect.

The research results show that the influence of the combination of behavioral agent type and information framing on green consumption intention is constrained by consumers’ interpretation level: low interpretation level consumers are more sensitive to the concrete and immediate benefits of individual behavior positive information, while high interpretation level consumers pay more attention to the long-term collective risks of group behavior negative information. This finding is consistent with the interpretation level consistency effect (Jin and He, 2013), that is, when the information presentation form is consistent with consumers’ cognitive processing mode, the information persuasion effect is the strongest. At the same time, the good reliability and validity of all scales and the effectiveness of variable manipulation ensure the scientificity and credibility of the research conclusions.

## 4 Conclusion and discussion

### 4.1 Research conclusion

This study integrates prospect theory, identity theory, and explanatory level theory, using social media videos as the research context to explore the impact of behavioral agency types on green purchase intention from the perspective of information framing. Through three experiments, the proposed research hypotheses were validated, leading to the following conclusions:

First, there is a significant interaction effect between the types of behavioral agents and information framing in social media content, which can synergistically influence consumers’ green consumption intention. Specifically, positive information matching individual green behavior, and negative information matching group green behavior can significantly enhance consumers’ green purchase intention. This finding breaks the single research perspective of existing literature on content characteristics, and reveals the matching effect between behavioral agent type and information framing in green communication.

Second, self-identity and social identity play a differentiated mediating role in the above interactive effect. Self-identity is the cognitive transmission path of “individual behavior+positive information”: positive information of individual behavior activates consumers’ environmental self-identity by highlighting the consistency between personal green behavior and self-values, and then promotes green consumption intention. Social identity is the cognitive transmission path of “group behavior+negative information”: negative information of group behavior enhances consumers’ sense of group belonging and environmental social responsibility, and social identity further translates this psychological perception into green consumption intention.

Third, consumers’ interpretation level has a significant moderating effect on the interaction between behavioral agent type and information framing, and the moderating effect conforms to the cognitive consistency principle. For low interpretation level consumers (focus on concrete details and immediate benefits), the combination of individual behavior and positive information has a significant promoting effect on green consumption intention, while the effect of group behavior with positive/negative information is not significant; for high interpretation level consumers (focus on abstract connotation and long-term impact), the combination of group behavior and negative information has a significant promoting effect, while the effect of individual behavior with positive/negative information is not significant.

### 4.2 Theoretical contribution

This study integrates three classic theories (prospect theory, identity theory, interpretation level theory) to construct a multi-variable synergistic influence model of “behavioral agent type-information framing-identity-interpretation level” on green consumption intention, which makes important theoretical contributions to the research field of green consumption and social media communication, and the specific contributions are as follows:

First, deepen the application of prospect theory in the context of social media green consumption, and expand the research boundary of framing effect. Centered on prospect theory, this study systematically analyzes the matching relationships and interactive mechanisms between behavioral agent types and information framing in social media videos, and empirically verifies that the framing effect is not a single effect, but is constrained by the behavioral agent type of information dissemination. The study clarifies the optimal matching mode of the two variables, and breaks the research limitation of existing literature that only focuses on the single effect of information framing or behavioral agent type, providing a new research perspective for the framing effect research in the digital context.

Secondly, established a differentiated identity mediation mechanism, and improve the theoretical framework of green consumption psychological mechanism. This study reveals that self-identity and social identity play a differentiated mediating role in the green consumption intention formation process, and clarifies the specific cognitive transmission paths of the two identity variables under different content characteristic combinations. This finding makes up for the research gap of existing literature that only confirms the predictive role of identity on green consumption behavior but does not explore its differentiated mediation mechanism, and enriches the theoretical research on the psychological mechanism of green consumption decision-making.

Third, expand the application scenarios of the Interpretation level theory, and identify the cognitive boundary condition of green communication effect. This study for the first time introduces interpretation level theory into the research of social media green communication, and verifies that consumers’ cognitive processing mode (interpretation level) is an important boundary condition of the interaction effect between behavioral agent type and information framing. The research results confirm the interpretation level consistency effect in the green consumption field, and expand the application scope of interpretation level theory in the consumer information communication field, providing a new cognitive perspective for understanding the heterogeneous response of consumers to green information.

### 4.3 Practical significance

The empirical findings of this study offer stratified and actionable strategies for social media marketing managers, advertising practitioners, and governmental environmental bodies.

#### 4.3.1 Precision matching of behavioral agents and information framing

Marketing practitioners should move beyond generic content by aligning the “who” (agent) with the “how” (frame) based on the specific green objective. For individual-level consumption (e.g., energy-saving appliances or eco-friendly tableware), a combination of “Individual Agent+Positive Framing” is most effective. By leveraging KOLs or peer testimonials to highlight personal gains-such as cost efficiency and enhanced quality of life-firms can activate self-interested motivations. Conversely, for collective environmental advocacy (e.g., waste sorting or public welfare projects), a “Group Agent+Negative Framing” approach should be prioritized. Depicting collective action alongside the consequences of ecological degradation creates a sense of urgency and social responsibility, effectively overcoming the “high awareness, low action” paradox in green marketing.

#### 4.3.2 Embedding and activating identity-based mechanisms

The persuasive power of social media content depends heavily on its ability to trigger identity recognition. In video production, “identity cues” should be strategically embedded. For individual-focused content, messaging should emphasize that green consumption is a reflection of a responsible lifestyle, using interactive features to help users link eco-friendly choices to their self-image. For group-focused content, the narrative should strengthen the “we-identity” (e.g., “Join the community of environmental protectors”). Utilizing collective social functions, such as group challenges or community discussions, can transform green intention into a proactive behavior aimed at maintaining social standing and belonging.

#### 4.3.3 Developing differentiated pathways based on construal levels

Our research confirms that a consumer’s cognitive abstraction (construal level) serves as a critical boundary for message effectiveness. For Low-Construal Consumers (e.g., mass audiences or elderly groups): Content should focus on the “how-to” and immediate utility. Using lightweight formats like short videos to demonstrate the low barriers and economic benefits of green habits can prevent the cognitive overload often caused by grand narratives. For High-Construal Consumers (e.g., environmental enthusiasts or highly educated groups): Communication should possess greater depth and logical sophistication, focusing on long-term ecological values and macro-visions like “Carbon Neutrality.” Furthermore, platforms can leverage algorithmic profiling to identify a user’s construal level based on browsing habits, enabling the precision delivery of green information and maximizing the efficiency of intention-to-behavior conversion.

#### 4.3.4 Optimizing public governance and social norm construction

Governmental bodies can utilize these insights to refine their environmental communication matrices. Public service announcements should utilize the “Individual+Gain” model to guide personal habits and the “Group+Loss” model to foster collective accountability. By embedding identity cues in public campaigns, policymakers can transition green consumption from an isolated individual choice into a self-evident social norm and a pursuit of personal value, ultimately elevating the environmental literacy of society at large.

### 4.4 Limitations and prospects

Although this study has obtained valuable research conclusions through rigorous experimental design and data analysis, there are still some limitations due to the constraints of research design and scenario settings:

First, the study faces limitations in research scenarios and stimulus materials. While this study used Bilibili platform videos as stimuli and focused on its user demographics, significant differences exist in demographic characteristics, usage habits, and content dissemination patterns across social media platforms. For instance, Weibo users tend to prioritize instant news and concise content, whereas WeChat users prefer lengthy articles and social sharing. These differences may influence how users perceive and interpret green information. Additionally, using stimuli from a single platform fails to capture the unique content ecosystems of different platforms, potentially limiting the external validity of research findings. Future studies should consider employing diverse stimuli that reflect the characteristics of various social media platforms. By comparing user behaviors across platforms, researchers can reveal how platform features affect the dissemination of green information and how different user groups respond to green consumption content. This approach not only enhances the external validity of research but also provides more targeted guidance for green marketing strategies in diverse social media environments.

Second, the dimensions of moderating variables require further exploration. While this study incorporated explanatory level as a moderating variable to examine its role in the “behavioral agency type-information framing” interaction effect, green consumption intention is shaped by multiple factors. Other cognitive and psychological variables may also play moderating roles. For instance, Laukkonen et al. (2023) demonstrated that insight can influence the ideas and information shared by others. Future research should investigate additional moderating variables affecting the green behavior-information framing interaction, including personal values, social influence, situational factors, and emotional responses. By developing a multi-dimensional moderating framework, researchers can gain comprehensive insights to help businesses design more effective green communication strategies. Additionally, consumers’ cultural background, education level, and socioeconomic status may significantly impact their green consumption intentions. Therefore, future studies should employ larger sample sizes and multidimensional analytical frameworks to explore behavioral differences across diverse contexts, providing more precise recommendations for green marketing.

## Data Availability

The raw data supporting the conclusions of this article will be made available by the authors, without undue reservation.
